# Longitudinal assessment of glymphatic changes following mild traumatic brain injury: Insights from perivascular space burden and DTI-ALPS imaging

**DOI:** 10.3389/fneur.2024.1443496

**Published:** 2024-08-07

**Authors:** Jiachen Zhuo, Prashant Raghavan, Jiang Li, Steven Roys, Rosy Linda Njonkou Tchoquessi, Hegang Chen, Emerson M. Wickwire, Gunjan Y. Parikh, Gary T. Schwartzbauer, Lynn M. Grattan, Ze Wang, Rao P. Gullapalli, Neeraj Badjatia

**Affiliations:** ^1^Center for Advanced Imaging Research, University of Maryland School of Medicine, Baltimore, MD, United States; ^2^Department of Diagnostic Radiology and Nuclear Medicine, University of Maryland School of Medicine, Baltimore, MD, United States; ^3^Department of Epidemiology and Public Health, University of Maryland School of Medicine, Baltimore, MD, United States; ^4^Department of Psychiatry and Medicine, University of Maryland School of Medicine, Baltimore, MD, United States; ^5^Program in Trauma, University of Maryland School of Medicine, Baltimore, MD, United States; ^6^Department of Neurology, University of Maryland School of Medicine, Baltimore, MD, United States; ^7^Department of Neurosurgery, University of Maryland School of Medicine, Baltimore, MD, United States

**Keywords:** traumatic brain injury, glymphatic system, perivascular space, diffusion tensor imaging, MRI, sleep disturbance, memory problem

## Abstract

**Introduction:**

Traumatic brain injury (TBI) even in the mild form may result in long-lasting post-concussion symptoms. TBI is also a known risk to late-life neurodegeneration. Recent studies suggest that dysfunction in the glymphatic system, responsible for clearing protein waste from the brain, may play a pivotal role in the development of dementia following TBI. Given the diverse nature of TBI, longitudinal investigations are essential to comprehending the dynamic changes in the glymphatic system and its implications for recovery.

**Methods:**

In this prospective study, we evaluated two promising glymphatic imaging markers, namely the enlarged perivascular space (ePVS) burden and Diffusion Tensor Imaging-based ALPS index, in 44 patients with mTBI at two early post-injury time points: approximately 14 days (14Day) and 6–12 months (6–12Mon) post-injury, while also examining their associations with post-concussion symptoms. Additionally, 37 controls, comprising both orthopedic patients and healthy individuals, were included for comparative analysis.

**Results:**

Our key findings include: (1) White matter ePVS burden (WM-ePVS) and ALPS index exhibit significant correlations with age. (2) Elevated WM-ePVS burden in acute mTBI (14Day) is significantly linked to a higher number of post-concussion symptoms, particularly memory problems. (3) The increase in the ALPS index from acute (14Day) to the chronic (6–12Mon) phases in mTBI patients correlates with improvement in sleep measures. Furthermore, incorporating WM-ePVS burden and the ALPS index from acute phase enhances the prediction of chronic memory problems beyond socio-demographic and basic clinical information.

**Conclusion:**

ePVS burden and ALPS index offers distinct values in assessing glymphatic structure and activity. Early evaluation of glymphatic function could be crucial for understanding TBI recovery and developing targeted interventions to improve patient outcomes.

## 1 Introduction

Traumatic brain injury (TBI) is a significant global health concern, affecting approximately 55 million people worldwide (1). About 70% of all TBI is categorized as mild, with admission Glasgow Coma Scale (GCS) of 13–15 (2). However, even “mild” brain injuries may result in structural and functional changes in the brain, leading to long term post-concussion symptoms (3–7). Among the most common post-concussion symptoms are cognitive issues, such as memory loss, reduced information processing speed, problems with divided attention, and decline in executive functions (8–10). TBI is also known as a risk factor in the development of neurodegenerative diseases later in life, such as Alzheimer’s Disease (11–14).

While it is still unclear how TBI contributes to progressive neurodegeneration, increasing evidence points toward glymphatic dysfunction and impaired protein waste clearance as potential key factors in developing dementia (15, 16). The glymphatic system, a structured fluid transport network, spans the periarterial and perivenous spaces (located in the perivascular space), as well as the brain’s interstitial spaces (17–19). This system facilitates the exchange of solutes between cerebrospinal fluid (CSF) and the interstitial fluid, allowing waste products from the brain to be transported away. The glymphatic system is also shown to be most active during sleep, when the brain’s extracellular space expands, allowing CSF infiltration along the perivascular space (20, 21). Notably, experimental TBI has been shown to cause a 60% reduction in glymphatic flow, which persists for over a month (22). Additional evidence from animal studies also supports the idea that glymphatic dysfunction contributes to subsequent neurodegeneration and cognitive decline (17, 23). Several studies have targeted novel treatments that consider the glymphatic system as an intervention target for TBI and neurodegeneration. For example, Lee et al. demonstrated that focused ultrasound can improve glymphatic-lymphatic drainage of amyloid beta in a mouse model of Alzheimer’s disease (24). Additionally, osteopathic manipulative treatment has been shown to optimize glial lymphatic clearance of CSF and improve interstitial waste product removal in severe TBI patients (25), as well as alleviate concussion symptoms (26).

Imaging the glymphatic system directly in humans poses significant challenges due to the necessity of intrathecally injecting gadolinium (Gd)-based contrast agents as CSF tracers to track their passage through the glymphatic system (27, 28). However, this method is invasive and not suitable for widespread application in the general population. Several non-invasive glymphatic imaging techniques have been developed (29, 30) as alternative approaches for assessing the glymphatic system. Among them, two most promising and broadly used methods are the structural MRI-based Enlarged PeriVascular Spaces (ePVS) method (31, 32), and the Diffusion Tensor Imaging ALong the Perivascular Spaces (DTI-ALPS) method (33, 34). Elevated ePVS burden, identified from T2-weighted MRI images or in junction with T1-weighted MRI images, could indicate blockage in interstitial fluid drainage (35) and impaired glymphatic function (31, 36). A high ePVS burden is linked to normal aging (37, 38), TBI (39–41), Alzheimer’s disease and related dementia (42, 43), and sleep quality (44, 45). Recent research has shown correlations between increased ePVS burden and poor sleep, as well as persistent post-concussion symptoms in TBI patients (39, 40, 46). The DTI ALPS index, derived from diffusion tensor imaging (DTI), quantifies glymphatic diffusion activity by assessing diffusivity along the deep medullary veins at the level of the lateral ventricular bodies (33). A higher ALPS index, indicative of increased glymphatic system activity, has been shown to correlate with better cognitive function and brain reserve in the aging population (47). Conversely, a lower ALPS index is linked to poor sleep (48, 49) and an elevated risk of developing Alzheimer’s disease and related dementia (33, 34, 47, 50, 51). In TBI patients, cross-sectional studies have yielded mixed results, with some reporting a reduced ALPS index post-injury (52–54) while others showing an increased index (55). This discrepancy may reflect the dynamic changes in white matter structure and glymphatic activity during TBI recovery.

Due to the heterogeneous nature of TBI and its association with various pre-existing risk factors impacting patient outcomes (2, 56, 57), longitudinal studies are critical for understanding the dynamic alterations in the glymphatic system and gaining insights into its role in TBI recovery. This study aims to investigate the longitudinal changes in glymphatic imaging markers, specifically ePVS burden and the DTI-ALPS index, in patients with mild TBI (mTBI) during the early post-injury phase and up to one year post-injury. We hypothesize that glymphatic imaging markers will correlate with patient symptom ratings, and that longitudinal changes in these markers will reflect underlying glymphatic alterations associated with TBI recovery. By elucidating these relationships, we hope to provide insights into the potential of glymphatic imaging markers as predictors of long-term outcomes in TBI patients.

## 2 Materials and methods

### 2.1 Participants

Participants in this study were prospectively recruited, which included mTBI patients, orthopedic control patients with no head injury, and healthy controls without mTBI or orthopedic injury. Age range for all participants was 18–60 year and included 58% male and 42% female. All participants were English speaking and had no contraindications for MRI. mTBI and orthopedic control patients were recruited from the R Adam Cowley Shock Trauma Center (STC) at the University of Maryland Medical Center. Inclusion criteria for the *mTBI patients* included (I1) an admission Glasgow Coma Score (GCS) of 13–15; (I2) a positive admission CT, or if negative CT, then with documented/reported loss of consciousness or amnesia and/or evidence of facial trauma; (I3) admission to the STC within 24 h of injury. Exclusion criteria included (E1) penetrating head injury; (E2) status post trauma due to asphyxiation; (E3) history of prior head injury requiring medical attention within the last 5 years, or prior injury at any time which resulted in persistent neurological or psychological deficits; (E4) history of neurological and/or psychiatric illness such as brain tumor, multiple sclerosis, Alzheimer’s disease, bipolar disorder, schizophrenia, seizures, etc.; (E5) history of stroke or myocardial infarction; (E6) active duty military status; (E7) police custody or prisoner status; and (E8) pregnant women. For the *orthopedic control group*, the inclusion criteria were being admitted to the STC for injuries other than brain injury and negative head CT if available, along with criteria (I3) as previously stated for mTBI patients. The same exclusion criteria applied. *Healthy controls* were recruited through online advertisements and flyers posted on the University of Maryland Baltimore campus, or they may be family or friends of the recruited patients. The inclusion criteria included no brain abnormalities, along with all the same exclusion criteria. The study was approved by the Institutional Review Board of the University of Maryland Baltimore. All participants underwent informed consent and were compensated for their participation in the study.

**Figure 1:**
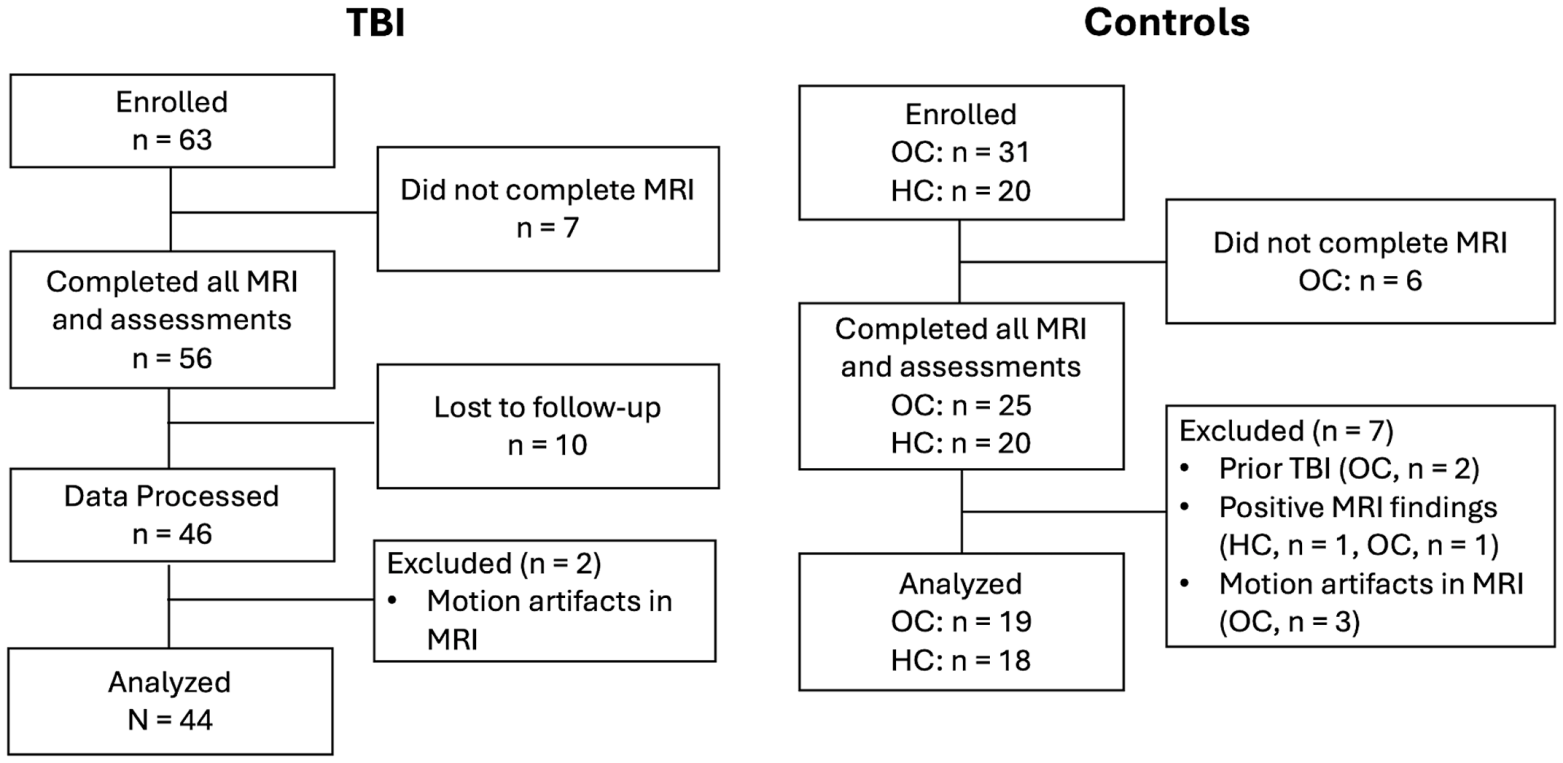
Flow diagram illustrating the progression of study participants from enrollment to data analysis. OC: Othopedic Control, HC: Healthy Control.

A total of 63 mTBI patients, 31 orthopedic control patients (OC) and 20 healthy controls (HC) were enrolled in the study. Figure 1 shows a flow diagram of the progression of study participants from enrollment to data analysis. 56 mTBI patients, 25 OCs and 20 HCs completed all MRI and assessments. For patients, their initial MRI visit was at approximately 14 days post their injury (14Day visit). 46 mTBI patients came back for follow-up visits at 6 months to 12 months (6–12Mon visit). Data from two additional patients were excluded after manual inspection of data quality due to motion artifacts. For controls, only one study visit was considered. 2 OCs were excluded due to later discovery of prior history of TBI, 1 HC and 1 OC were excluded due to clinically relevant incidental findings from MRI scans, 3 OCs were excluded due to motion artifacts. Eventually, a total of 44 mTBI and 37 controls (19 OCs and 18 HCs) were included in our data analysis.

For all mTBI patients, their admission CT findings were obtained from electronic medical database. MRI imaging findings were based on radiology reads from the 14Day conventional MRI sequences (T1-weighted T2-weighted, FLAIR, SWI, and diffusion weighted images). Any findings from either the admission CT or 14Day MRI were considered as positive for “CT or MRI findings.” No microbleeds were reported in regions assessed for DTI-ALPS index.

### 2.2 Behavioral assessments

All socio-demographic information was based on participant self-report. Participants also completed self-reported symptoms on the Modified Rivermead Post-Concussion Symptoms Questionnaire (RPQ) (58) and the Pittsburgh Sleep Quality Index (PSQI) (59) at each study visit. RPQ covers 22 symptoms, each is rated through a 5-point ordinal scale: 0 = not experienced at all, 1 = no more of a problem, 2 = a mild problem, 3 = a moderate problem, and 4 = a severe problem. Note that while patients were directed to report symptoms arising after the injury, healthy controls were directed to report symptoms as they experienced in the past month to establish a prevalence of these symptoms in the general adult population. Patients reporting at least a mild symptom (RPQ rating ≥ 2) were considered symptomatic, similar as in the TRACK-TBI study (10). Total RPQ scores were calculated, along with total number of symptoms (rated at least mild). The Pittsburgh Sleep Quality Index (PSQI) is a 19-item questionnaire that assesses the quality and patterns of sleep. This measure focuses on the participants’ usual sleep habits during the past month and has been previously used in studies of mTBI (40, 60, 61).

### 2.3 MRI data acquisition

All MRIs were acquired in a 3 T Siemens Prisma scanner with a 64-channel head and neck coil. Imaging protocol included 3D T1-weighted MPRAGE, 3D T2-weighted SPACE, and diffusion-weighted MRI. Both T1w and T2w images were acquired with 1mm^3^ isotropic resolution, 256 mm FOV, and 176 sagittal sections. Other imaging parameters for T1w were TE/TR/TI = 3.37 ms/4000 ms/1400 ms, flip angle = 6^0^, BW = 200 Hz/Px, iPAT factor of 2; and for T2w were TE/TR = 349 ms/3200 ms, Turbo factor 280, BW = 781 Hz/Px, iPAT factor of 2×2. Diffusion MRIs were acquired with a 3× simultaneous multi-slice (SMS) EPI sequence in 75 axial slices with FOV = 224 mm, isotropic resolution of 2 mm, TE/TR = 78 ms/3500 ms, 2 b = 0 s/mm^2^ volumes and 45 diffusion directions at b = 1000s/mm^2^ and 90 diffusion directions at b = 2,500 s/mm^2^. An additional B0 volume was acquired with reversed phase-encoding direction to correct for susceptibility artifacts.

### 2.4 Image process

#### 2.4.1 Enlarged PVS (ePVS) burden

We employed a previously described method for automatic segmentation of the ePVS burden using 3D T1w and T2w images (32, 46). Briefly speaking, the 3D T1w and T2w images were first processed with the Human Connectome Project (HCP) pipeline (62) for rigidly aligning both sets of images to the MNI space, up-sampled to 0.7 mm resolution, followed with sub-cortical segmentation using the FreeSurfer software (version v6.0.1). Following intensity normalization based on white matter signal, enhanced perivascular space contrast (EPC) image was generated, followed with frangi filter to create a vesselness map. To create the final binary ePVS mask, a threshold ‘h’ of 0.0000002 combined with a minimum cluster size of 9 (~3mm^3^) was used for white matter ePVS. A slightly lower h was used here than previously noted (32, 46) as we found this combined with a minimum cluster size in our data provided the best balance between sensitivity in detecting ePVS and limiting false detection rate (46). For the basal ganglia (BG) region, h was set at 0.0000003 with the same minimum cluster size.

**Figure 2:**
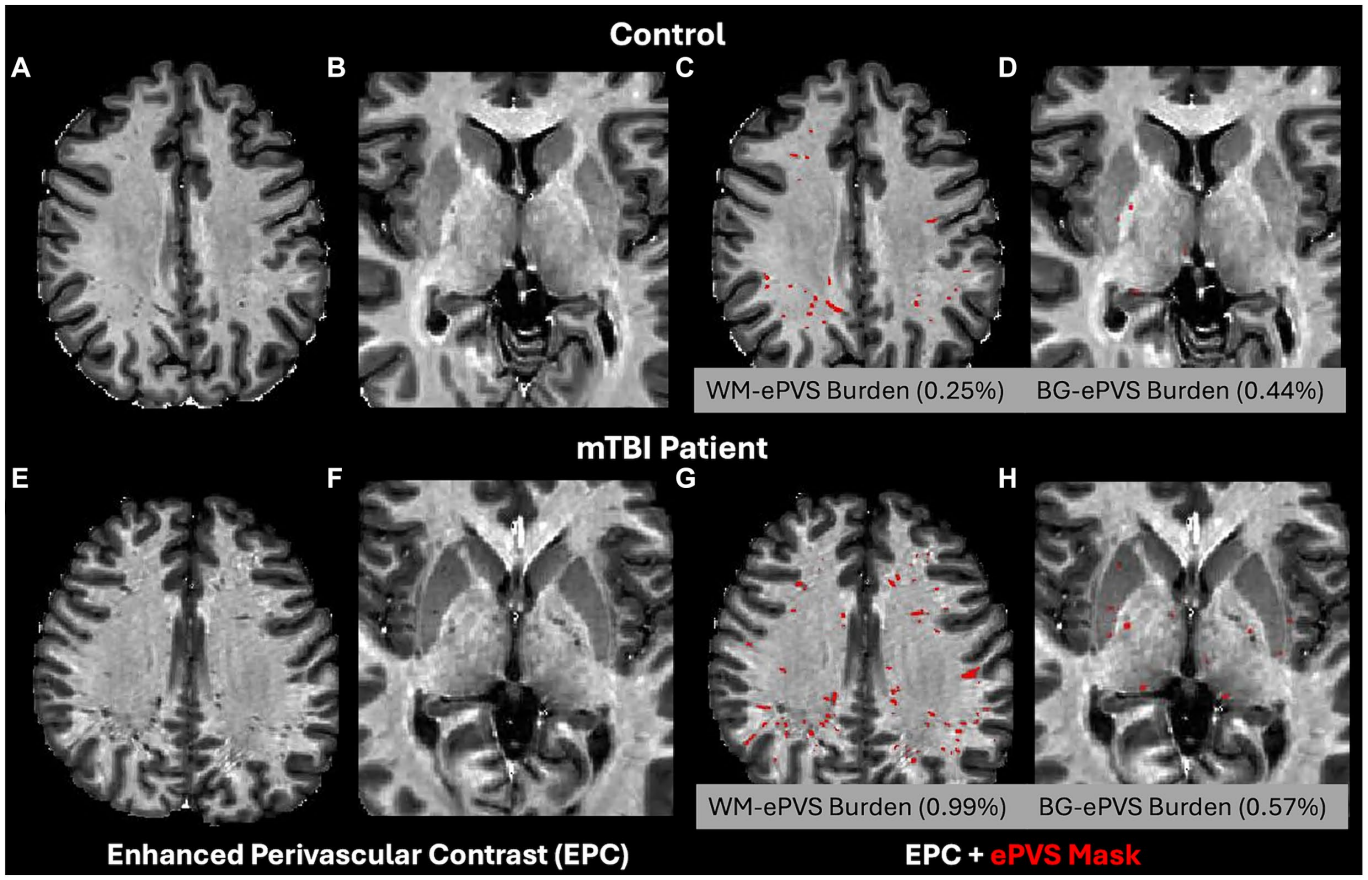
Enhanced Perivascular Contrast (EPC) images and segmented ePVS masks (in red) on a healthy control and TBI patients, of similar age between 20–30 years. **(A,C,E,G)** ePVS segmentation in whole brain white matter (WM). **(B,D,F,H)** ePVS segmentation in the basal ganglia (BG) region. Calculated WM-ePVS burden and the BG-ePVS burden were also shown for the corresponding cases.

ePVS volume was calculated from both the whole brain WM and BG regions based on the FreeSurfer segmented masks. To account for brain size differences, the ePVS burden (46) was calculated as *ePVS burden = 100% × ePVS volume/region volume.* The corresponding region was either the whole brain white matter (WM-ePVS burden) or basal ganglia (BG-ePVS burden). Figure 2 shows an example of the EPC images along with segmented ePVS masks on a healthy control and an mTBI patient of similar ages, for both the white matter regions and basal ganglia regions, along with calculated WM-ePVS burden and BG-ePVS burden. Notably due to the lower contrast between ePVS and gray matter in the basal ganglia regions, the BG-ePVS segmentation appeared to be less reliable than WM-ePVS, with some ePVS missing.

#### 2.4.2 DTI-ALPS index

**Figure 3:**
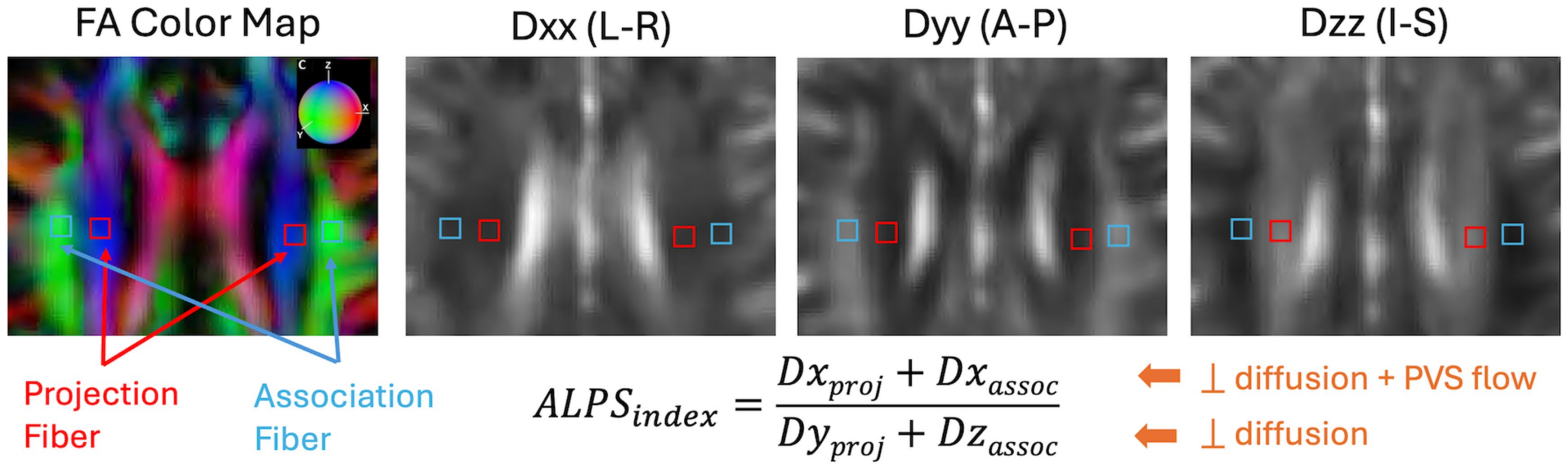
Representation and calculation of the DTI-ALPS index are shown on an example subject from the projection fiber (Dx_proj_ and Dy_proj_) and the association fiber (Dx_assoc_ and Dz_assoc_). Dxx, Dyy, and Dzz are diffusion coefficient maps along the X-, Y-, and Z-directions. The color sphere in the FA color map indicates the color coding of the diffusion tensor. L-R, Left–Right; A-P, Anterior–Posterior; I-S, Inferior–Superior.

DTI-ALPS index was calculated using the method originally proposed (33). All diffusion-weighted images were preprocessed using the MRtrix3 software package (63). Preprocessing steps included denoising (64), eddy-current correction and motion correction (65), and bias field correction using the N4 algorithm as provided in ANTs (66). Diffusion Kurtosis Imaging reconstruction were then performed using in-house Matlab program (67, 68) to estimate both a diffusion tensor and a kurtosis tensor. The diffusion tensor was then used to generate the diffusion coefficient maps along the X- (Dxx), Y- (Dyy), and Z- (Dzz) directions. For regions-of-interest (ROI) analysis, to improve reproducibility in ALPS calculation (69), we took the approach to first warp all the diffusion maps to the MNI152 standard space provided in FSL (FMRIB Software Library, version 6.0, Oxford, UK) (70). The B0 image was first registered to the HCP-processed T1w images using the EPI to structural registration tool, epi_reg (part of FSL). T1w images were warped to the MNI152 space using the SyN tool (71) in ANTs. All registration steps were concatenated first before warping the diffusion maps to the MNI152 space. Additionally, the principal diffusion direction map was warped using the vecreg tool (part of FDT-FMRIB’s Diffusion Toolbox) to create colored FA map in the MNI152 space. Figure 3 shows the representative FA colormap and diffusivity maps on a representative participant in the MNI152 space, where a single set of four 16mm^2^ square ROIs were manually drawn in the projection fiber and association fiber adjacent to the medullary veins at the level of the lateral ventricle body. Placement of the ROIs were checked on all subjects and manually adjusted (if needed) to ensure proper coverage within the projection and association fiber regions. Mean values from the two bi-lateral ROIs were extracted for ALPS index calculation.

### 2.5 Data analysis

Glymphatic imaging markers included WM-ePVS burden, BG-ePVS burden and ALPS index. Participant socio-demographic information included age, sex, years of education. Patient clinical information included admission GCS, binary indicator of positive CT or MRI findings (based on admission CT or 14Day-MRI). Participant assessment scores included total RPQ score for symptoms (range: 0–88), individual symptom score (0–4), total number of symptoms (0–22), and PSQI scores for sleep quality (0–21).

We first tested for differences of imaging markers and symptom scores between the two control cohorts (OCs and HCs) using two-sample t-tests for continuous variables and chi-squared tests for categorical variables. When no significant differences were found, we combined the two control cohorts into a single control group. We sub-grouped mTBI patients into those with and without memory problems based on their 14Day memory symptoms (RPQ rating ≥ 2). Group comparisons (controls vs. mTBI, mTBI- Memory Prob vs. mTBI-No Memory Prob) were conducted two-sample t-tests and chi-squared tests.

Associations between imaging markers and age were analyzed using Pearson correlations. Associations of imaging measures with assessment scores were analyzed using multiple variable linear regression with age and sex as covariables. Longitudinal analysis in mTBI patients was conducted using linear mixed model with visit (14Day, 6–12Mon) as fixed effect, patient as a random effect, and age and sex as covariates. The ratio of imaging markers and assessment scores (6–12Mon/14Day) were calculated to examine longitudinal changes over time.

Logistic regression with stepwise selection was used to develop prediction models for binary memory problems (RPQ rating ≥ 2) at the 6–12Mon visit, including socio-demographic (Age, Sex, Education), clinical information (GCS, CT or MRI findings), and glymphatic imaging markers (WM-ePVS, BG-ePVS, ALPS index) at the 14Day visit as independent variables. Receiver Operating Characteristic (ROC) curves were generated to compare the basic model (socio-demographic and clinical information) with the model including 14Day glymphatic imaging markers. Model comparisons were visualized using ROC curves, and area under the curves (AUC) were compared using the bootstrap method.

All statistical analyses were performed in R version 4.3.0 (R Core Team, 2021). Multiple comparisons were corrected by the False Discovery Rate (FDR) method. The significance level was defined as *p* < 0.05.

## 3 Results

### 3.1 Orthopedic control (OC) vs. healthy control (HC)

Between-groups differences in socio-demographic, symptom ratings and glymphatic imaging markers between the orthopedic control and the healthy controls are presented in Table 1. There were no significant differences in age, any symptom ratings, or any glymphatic imaging markers. HCs did include more females (*p* = 0.043) and had significantly higher education years (*p* = 0.0003). When combined to a single control cohort, the combined sex and education level did not significantly differ than the mTBI cohort (Table 2). We therefore combined the OCs and HCs to increase the sample size for controls.

**Table 1:** Socio-demographic, symptoms, and imaging measures for the two control groups (orthopedic control, OC, and healthy controls, HC).

	Orthopedic control	Healthy control	*p*-value (OC vs. HC)
*N*	19	18	
Age (yrs)	37.3 ± 13.8 (19–60)	35.5 ± 12.3 (18–56)	0.79
Sex (Male)	12 (63%)	5 (28%)	0.043*
Education (yrs)	13.4 ± 1.9 (11–18)	17.1 ± 3.4 (10–24)	0.0003*
Race (W/B/A/O)	7/10/1/1	9/7/2/0	0.55
Body Mass Index (BMI)	28.3 ± 6.7 (20.7–53.7)	28.7 ± 9.8 (19.5–53.7)	0.87
Days post injury	13.8 ± 6.9 (6–29)	N/A	
Symptom ratings			
PSQI	7.4 ± 5.1 (0–17)	5.2 ± 3.5 (1–12)	0.17
RPQ	14.2 ± 15.1 (0–48)	8.6 ± 7.9 (0–26)	0.22
Total number of symptoms	4.3 ± 4.8 (0–14)	2.0 ± 2.4 (0–8)	0.12
Glymphatic imaging markers			
WM-ePVS (%)	0.40 ± 0.18	0.39 ± 0.18	0.90
BG-PVS (%)	0.48 ± 0.21	0.59 ± 0.22	0.13
ALPS Index	1.49 ± 0.14	1.51 ± 0.10	0.59

Values shown are Mean ± SD, with ranges or percentage in (). Race includes White (W), Black or African American (B), Asian (A), and Other (O). PSQI is the global PSQI score using the Pittsburgh Sleep Quality Questionnaire. RPQ is the total scores from the Rivermead Post Concussion Symptoms questionnaire. Total number of symptoms was counted when reported as at least mild symptoms (RPQ ratings ≥2). WM-ePVS is the white matter ePVS burden. BG-PVS is the basal ganglia ePVS burden. Significant *p*-values (*p* < 0.05) were shown in bold with *. MVC, Motor Vehicle Collision.

**Table 2:** Socio-demographic, symptoms, and imaging measures for control and mTBI patients at two visits (14Day and 6–12Mon).

	Control	mTBI		*p*-value (Control vs. mTBI)	
*N*	37	44			
Age (yrs)	36.7 ± 12.9 (18–60)	34.5 ± 11.3 (18–59)		0.41	
Sex (Male)	17 (38%)	24 (55%)		0.51	
Education (yrs)	15.2 ± 3.3 (10–24)	14.2 ± 2.8 (9–24)		0.17	
Race (W/B/A/O)	16/17/3/1	18/17/2/7		0.23	
Body Mass Index (BMI)	28.5 ± 8.2 (19.5–53.7)	27.6 ± 6.0 (17.4–42.7)		0.56	
Positive CT or MRI	0 (0%)	20 (45%)			
Admission GCS	N/A	14.7 ± 0.5 (13–15)			
Injury mechanism					
MVC		28 (64%)			
Assault		4 (9%)			
Fall		8 (18%)			
Sports/Recreational		4 (9%)			
		14Day	6–12Mon	14Day	6–12Mon
Days post injury	N/A	13.8 ± 8.1	306 ± 97		
		(2–45)	(157–508)		
Symptom ratings					
PSQI	6.5 ± 4.5 (0–17)	6.1 ± 3.0 (1–14)	6.9 ± 3.5 (0–14)	0.72	0.67
RPQ	11.8 ± 12.6 (0–48)	21.4 ± 18.4 (0–85)	21.7 ± 16.5 (0–73)	0.014*	0.006*
Total number of symptoms	3.3 ± 4.0 (0–14)	7.1 ± 5.5 (0–21)	6.8 ± 6.2 (0–22)	0.0015*	0.0064*
Glymphatic imaging markers					
WM-ePVS (%)	0.40 ± 0.18	0.35 ± 0.26	0.38 ± 0.30	0.38	0.68
BG-PVS (%)	0.53 ± 0.22	0.50 ± 0.30	0.60 ± 0.41	0.53	0.40
ALPS Index	1.51 ± 0.12	1.52 ± 0.13	1.50 ± 0.13	0.66	0.82

Values shown are Mean ± SD, with ranges or percentage in (). Positive CT or MRI findings are based on admission CT and clinical MRI sequences. Race includes White (W), Black or African American (B), Asian (A), and Other (O). PSQI is the global PSQI score using the Pittsburgh Sleep Quality Questionnaire. RPQ is the total scores from the Rivermead Post Concussion Symptoms questionnaire. Total number of symptoms was counted when reported as at least mild symptoms (RPQ ratings ≥2). WM-ePVS is the white matter ePVS burden. BG-PVS is the basal ganglia ePVS burden. Significant *p*-values (*p* < 0.05) were shown in bold with*. MVC, Motor Vehicle Collision.

### 3.2 Control vs. mTBI (14Day and 6–12Mon)

There were no differences in age, sex, education years, race, Body Mass Index (BMI) between the mTBI patients and the controls. 45% of mTBI patients had either a positive finding from their admission CT or Day14 MRI. The mean days-post-injury for the two visits were 13.8 days (Day14) and 306 days (6–12Mon).

Interestingly, mTBI patients had similar PSQI scores as the controls at both visits, indicating that sleep problems may be a prevalent issue even in the general adult population. mTBI patients had significantly higher RPQ scores (Day14: *p* = 0.014; 6–12Mon: *p* = 0.006) and total number of symptoms (Day14: *p* = 0.0015; 6–12Mon: *p* = 0.0064) than the controls, as expected.

**Figure 4:**
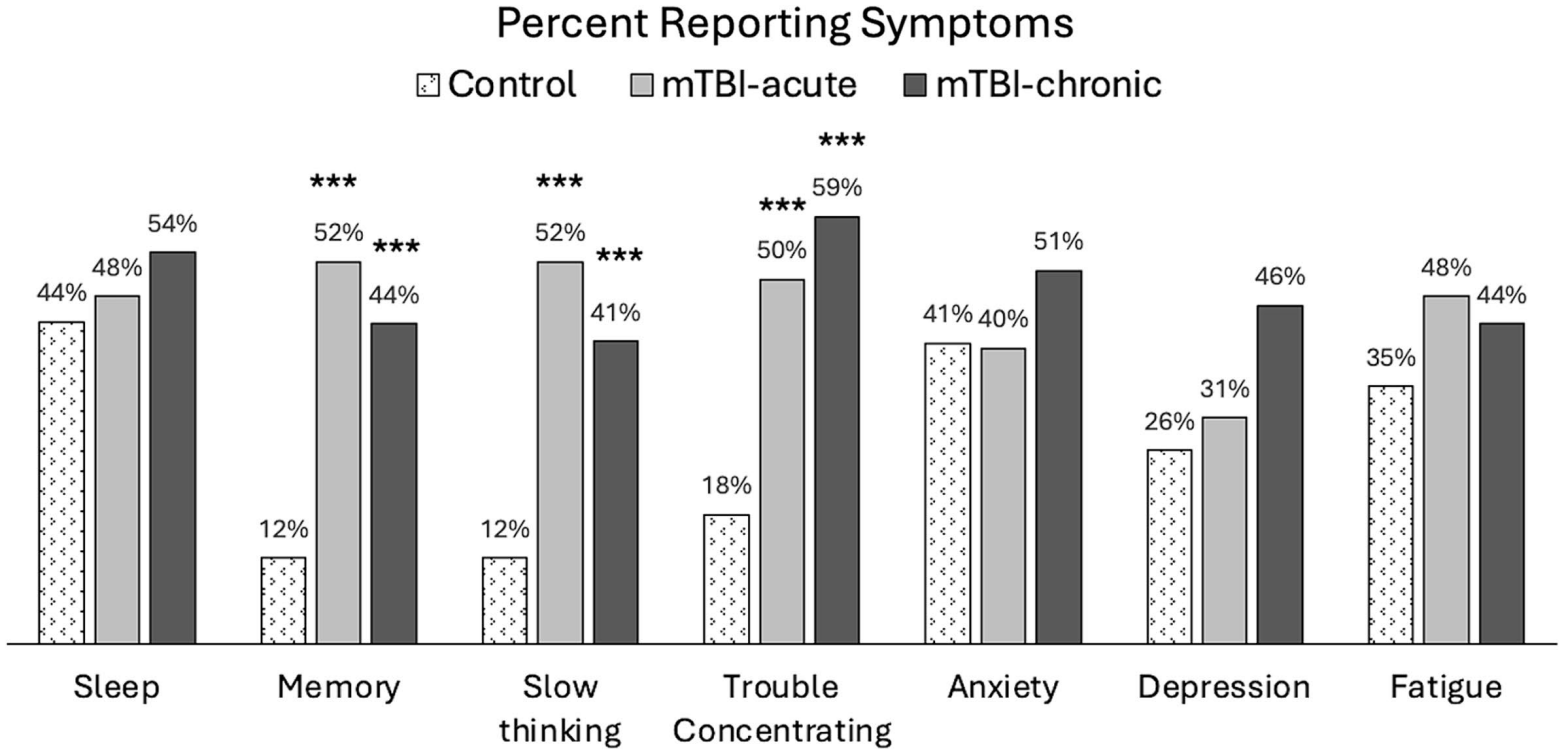
Percent population reporting at least mild symptoms (RPQ ratings ≥2) in controls and mTBI patients. Group comparisons were made between mTBI patients and controls using chi-square tests. ***p < 0.0005.

The following symptoms were found to persist in mTBI patients at the 6–12Mon visit: sleep (54%), memory problems (44%), slow thinking (41%), trouble concentrating (59%), anxiety (51%), depression (46%), and fatigue (44%), where more than 40% of patients were still reporting at least a mild problem (RPQ rating ≥ 2). Figure 4 shows prevalence of these symptoms for all groups at 6–12Mon. A significantly higher percentage of mTBI patients reported problems with cognitive symptoms, such as memory problems (*p* < 0.005), slow thinking (*p* < 0.005), and trouble concentrating (*p* < 0.005), but no significant differences were observed in other symptoms (sleep, anxiety, depression, or fatigue).

No significant differences were observed in the glymphatic imaging markers between the controls and mTBI patients at either the Day14 or the 6–12Mon visit.

### 3.3 Effect of age of glymphatic imaging markers

**Figure 5:**
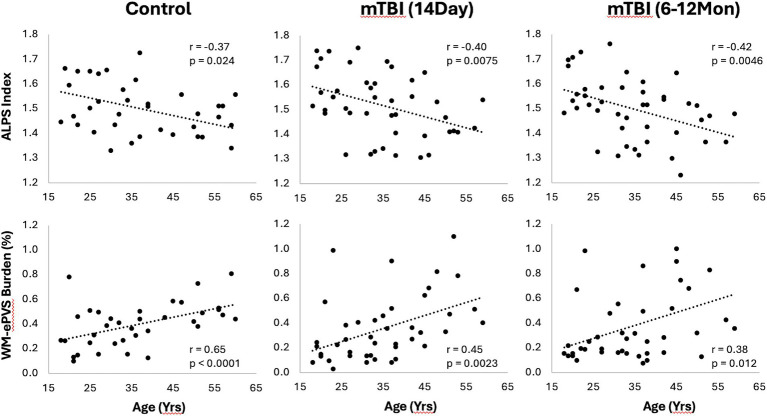
Scatter plots of ALPS index and WM-ePVS burden with age in controls and mTBI patients at both visits. Pearson correlation coefficient, *r*, and *p*-value were also shown for each group.

Both WM-ePVS burden and ALPS index were found to be significantly correlated with age for both controls and mTBI patients. Figure 5 shows the scattered plots of the ALPS index and WM-ePVS burden with age in control and mTBI patients at both visits. In all groups, ALPS index significantly decreases with age (Control: *r* = −0.37, *p* = 0.024; mTBI 14Day: *r* = −0.40, *p* = 0.0075; mTBI 6–12Mon: *r* = −0.42, *p* = 0.0045) while the WM-ePVS burden significantly increases with age (Control: *r* = 0.65, *p* < 0.0001; mTBI 14Day: *r* = 0.45, *p* = 0.0023; mTBI 6–12Mon: *r* = 0.38, *p* = 0.012). The age-slope is however not significantly different in mTBI patients at each visit as compared to controls. Noticeably the variability of imaging markers is higher in mTBI patients, especially in WM-ePVS burden where more outlier high WM-ePVS were spotted in mTBI patients. One of the outlier cases (mTBI 14Day, Age 23 yrs., WM-ePVS burden = 0.99%) was represented in Figure 2. No significant age-correlations were found for BG-ePVS burden (Control: *p* = 0.53, mTBI Day14: *p* = 0.77, mTBI 6–12Mon: *p* = 0.12).

### 3.4 Correlations between the glymphatic imaging markers

We also tested correlations between the glymphatic imaging markers among themselves for controls and mTBI at both visits. In controls, WM-ePVS burden was found to be burden was found to be positively correlated with the BG-ePVS burden (*r* = 0.34, *p* = 0.044), and negatively correlated with ALPS index (*r* = −0.36, *p* = 0.028). However, no significant correlations were found between these imaging markers in mTBI patients at any visit.

### 3.5 Association of glymphatic imaging markers with symptoms

No significant correlations were found between glymphatic imaging markers with any symptom ratings in controls, or mTBI patients at the 6–12Mon visit.

**Figure 6:**
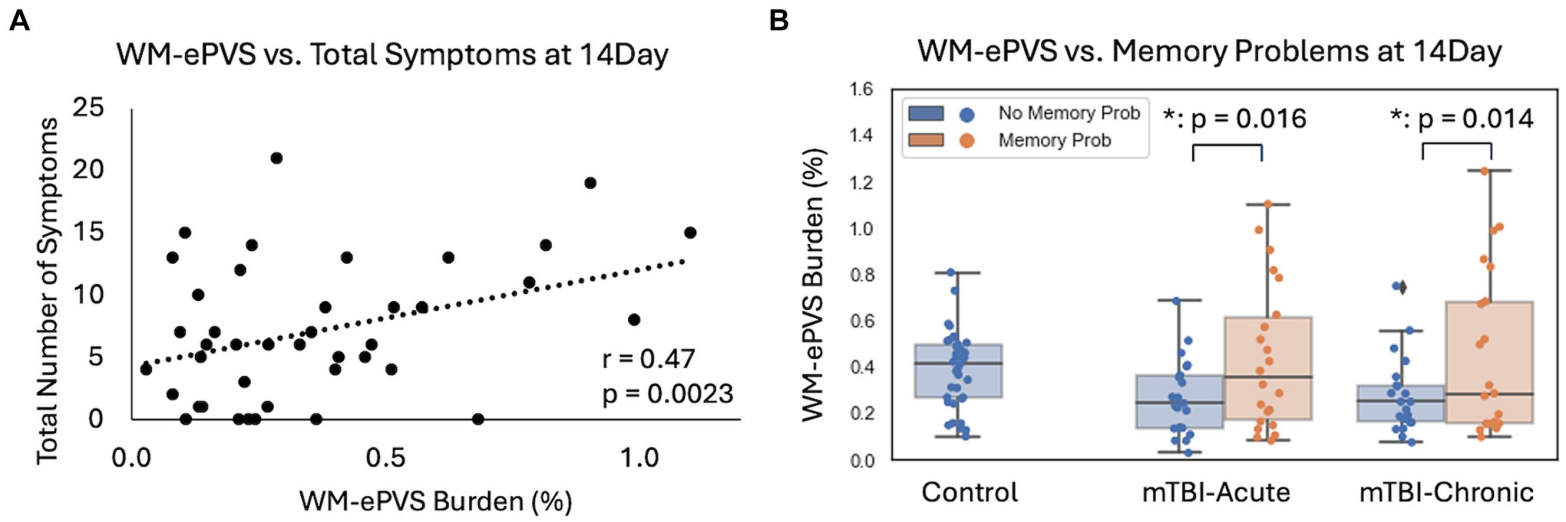
Associations of WM-ePVS burden with symptoms at 14Day in mTBI patients. **(A)** Scatter plots of WM-ePVS Burden and the total numbers of symptoms (with RPQ ratings ≥2) at 14Day, with age and sex-adjusted partial correlation coefficient and *p*-value. **(B)** WM-ePVS Burden in control and mTBI patients grouped by their reported memory problems (RPQ memory rating ≥ 2) at the 14Day visit.

For mTBI patients at the 14Day visit, the WM-ePVS burden were significantly correlated with the total number of symptoms (*p* = 0.0023) (Figure 6A), and more specifically memory problems (*p* = 0.017). We further divided mTBI patients based on their memory problems at the 14Day visit (RPQ memory ratings of ≥2), to two sub-groups: “No Memory Prob” and “With Memory Prob” (Table 3; Figure 6B). The two patient sub-groups had no significant differences in age, sex, education, days post injury, or the PSQI scores. Patients with memory problems do present with significantly higher RPQ score (*p* < 0.0001) and symptom number (*p* < 0.0001). The WM-ePVS burden was significantly higher in the “With Memory Prob” group than the “No Memory Prob” group (*p* = 0.016). ALPS indexes do not differ between the two groups. Following up to the 6–12Mon visit, these two mTBI patient sub-groups continue to have significantly different WM-ePVS burden (*p* = 0.014), even though 10 patients had improving memory symptoms, while another 7 had worsening memory symptoms. The “With Memory Prob” group also continue to have significantly higher RPQ score (*p* = 0.02) and symptom number (*p* = 0.004), although the differences were smaller, likely indicating a recovery. No differences in ALPS index were observed.

**Table 3:** Sicio-demographic, symptoms, and imaging measures for mTBI patients with and without memory problems at two visits (14Day and 6–12Mon).

	No Memory Prob at 14Day		With Memory Prob at 14Day		Group Differences (*p*-value)	
N	21		23			
Age (yrs)	35.3 ± 11.2 (19 – 59)		33.9 ± 11.5 (18 – 53)		0.68	
Sex (Male)	13 (62%)		11 (48%)		0.35	
Education (yrs)	14.4 ± 3.2 (9 – 24)		14.1 ± 2.4 (9 – 18)		0.74	
Race (W/B/A/O)	9/8/2/0		9/9/0/5		0.35	
BMI	27.1 ± 4.8 (17.6 – 40.3)		28.0 ± 7.1 (17.4 – 42.7)		0.65	
Positive CT or MRI	13 (62%)		7 (30%)		0.067	
Admission GCS	14.7 ± 0.7 (13 – 15)		14.9 ± 0.4 (14 – 15)		0.25	
Injury mechanism					0.58	
MVC	11 (52%)		17 (74%)			
Assault	2 (10%)		2 (9%)			
Fall	5 (24%)		3 (13%)			
Sports/recreational	3 (14%)		1 (4%)			
	14Day	6-12Mon	14Day	6-12Mon	14Day	6-12Mon
Days post injury	12.8 ± 9.1 (2 – 45)	14.6 ± 7.1 (3 – 34)	306 ± 99 (157 – 440)	305 ± 97 (173 – 508)	0.47	0.95
Symptom ratings
PSQI	5.7 ± 3.2 (1 – 14)	6.7 ± 4.0 (0 – 14)	6.5 ± 2.8 (3 – 13)	6.5 ± 2.8 (3 – 14)	0.42	0.72
RPQ	7.8 ± 7.1 (0 – 21)	15.1 ± 15.6 (0 – 46)	33.8 ± 16.7 (15 – 85)	27.2 ± 15.4 (4 – 73)	< 0.0001*	0.02*
Total Number of Symptoms	2.7 ± 2.5 (0 – 7)	4.0 ± 4.8 (0 – 15)	11.0 ± 4.3 (6 – 21)	9.3 ± 6.3 (1 – 22)	< 0.0001*	0.004*
Glymphatic imaging markers
WM-ePVS (%)	0.27 ± 0.16	0.28 ± 0.17	0.44 ± 0.31	0.47 ± 0.36	0.016*	0.014*
BG-PVS (%)	0.43 ± 0.27	0.53 ± 0.32	0.56 ± 0.32	0.76 ± 0.64	0.17	0.14
ALPS Index	1.53 ± 0.13	1.49 ± 0.14	1.50 ± 0.13	1.50 ± 0.12	0.44	0.82

The existence of memory problems was defined as RPQ ratings ≥ 2 at the 14Day visit. Values shown are Mean ± SD, with ranges or percentage in ( ). Positive CT or MRI findings are based on admission CT and clinical MRI sequences. Race includes White (W), Black or African American (B), Asian (A), and Other (O). PSQI is the global PSQI score using the Pittsburgh Sleep Quality Questionnaire. RPQ is the total scores from the Rivermead Post Concussion Symptoms questionnaire. Total Number of Symptoms was counted when reported as at least mild symptoms (RPQ ratings ≥ 2). WM-ePVS is the white matter ePVS burden. BG-PVS is the basal ganglia ePVS burden. Same mTBI patients were included for both visits, therefore demographic information stays the same. Significant *p*-values (*p* < 0.05) were shown in bold with *. BMI, Body Mass Index. MVC, Motor Vehicle Collision.

### 3.6 Longitudinal changes

No significant longitudinal differences were observed in mTBI patients from 14Day to 6–12Mon in PSQI, RPQ score or symptom numbers. The BG-ePVS burden significantly increased over time (*p* = 0.013), while other imaging markers were unchanged.

**Figure 7:**
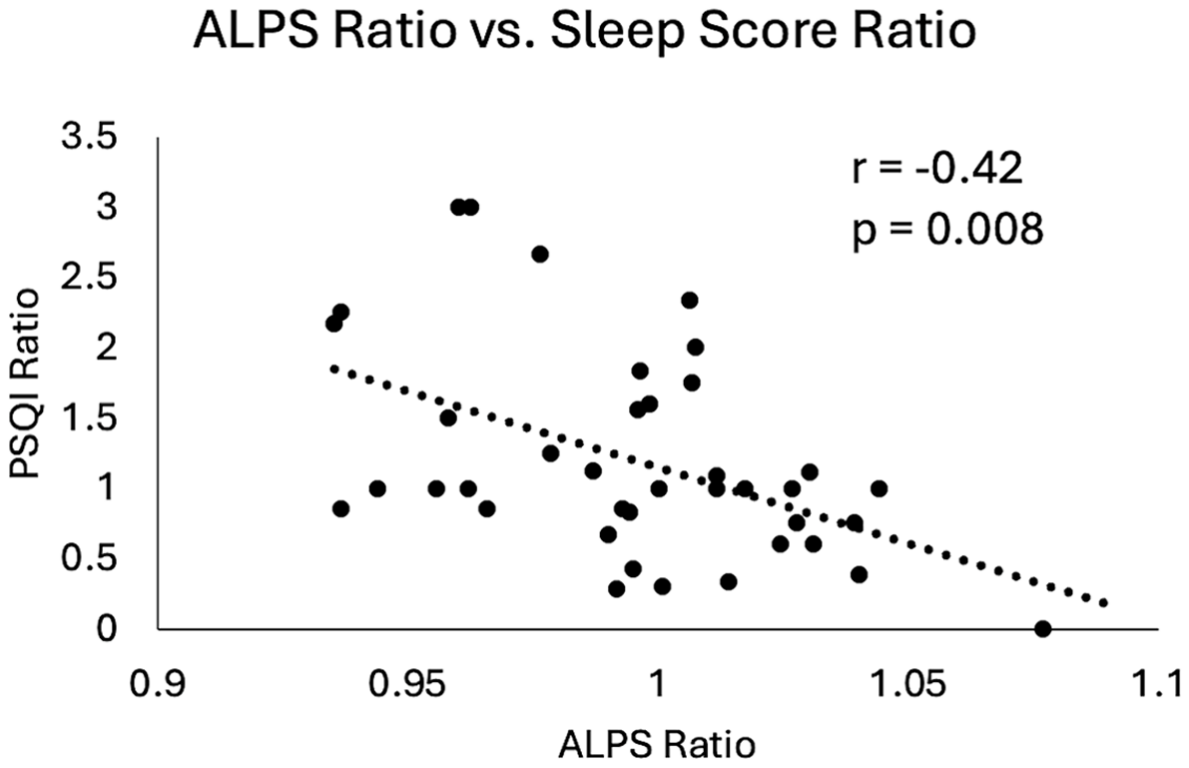
Scatter plot of changes in ALPS index and changes in sleep scores in mTBI patients from 14Day to 6–12Mon. Both the ALPS ratio and the PSQI ratio were calculated by dividing the 6–12Mon values by the 14Day values. Pearson correlation coefficient and *p*-value were displayed here.

When investigating the visit effect and symptom ratings for imaging markers, the ALPS index demonstrated a significant positive association with the global PSQI score (*p* = 0.0062). Furthermore, the ratios of the ALPS index and the ratio of the PSQI score (6–12Mon/14Day) were also significantly correlated (*r* = −0.42, *p* = 0.008) (Figure 7). Higher ALPS ratio, indicative of increase in the ALPS index from 14Day to 6–12Mon (increase glymphatic activity), was associated with reduction in PSQI scores (improvement in sleep condition).

### 3.7 Prediction of 6–12Mon memory problem

**Figure 8:**
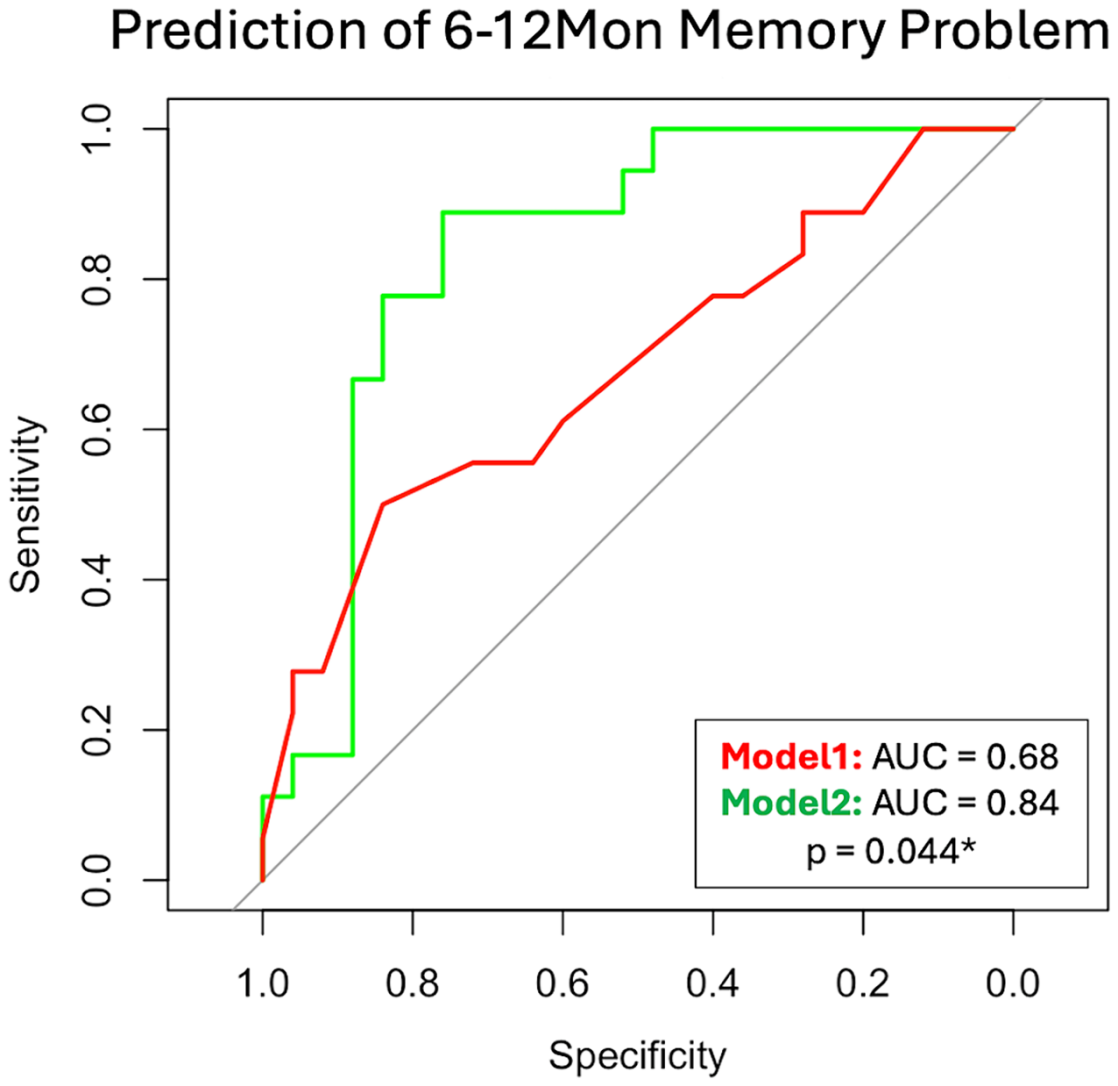
ROC curve for two logistic regression models predicting patient memory problems at the 6–12Mon visit (defined as RPQ rating ≥ 2). Model 1 used socio-demographic and clinical information, while Model 2 added 14Day glymphatic imaging measures. Both models were developed using step-wise selection. Model 1 included Education and CT or MRI findings, whereas Model 2 included Education, CT or MRI findings, WM-ePVS, and ALPS. The models were compared using the bootstrap method.

In the mTBI patient cohort, we explored two models to predict their 6–12Mon memory outcome, based on existence of their memory problems. The two models were Model 1: only with patients’ socio-demographic (Age, Sex, Education) and clinical information at 14Day (admission GCS, and CT or MRI findings). This represents the scenario for patients with basic clinical information and admission imaging findings. Model 2 included all parameters in Model 1 and the glymphatic imaging markers at 14Day (WM-ePVS, BG-ePVS and ALPS). Stepwise selection selected two parameters for Model 1: Education and CT or MRI findings; and four parameters for Model 2: Education, CT or MRI findings, WM-ePVS and ALPS. The coefficients for Model 2 are shown in Table 4. The ROC curve for Model 2 as compared to Model 1 was shown in Figure 8, with AUCs of 0.68 for Model 1 and 0.84 for Model 2. Adding WM-ePVS and ALPS measured at 14Day in Model 2 significantly improved model prediction for 6–12Mon memory problems (*p* = 0.044).

**Table 4:** Logistic regression model coefficients for Model 2 (Figure 7).

Coefficients	Estimate Std.	Error	Z value	Pr(>|z|)
(Intercept)	−10.4160	5.3262	−1.956	0.0505
Education	−0.2367	0.1184	−1.999	0.0456
CT_or_MRI (Yes)	−1.6464	0.8532	−1.930	0.0536
WM-ePVS	3.5513	1.7045	2.084	0.0372*
APLS	9.3073	3.8718	2.404	0.0162*

Among all socio-demographic and clinical information, Education and CT or MRI findings were selected to compose the best logistic model for prediction of 6–12Mon memory problem. 14Day WM-ePVS and ALPS were also significant factors (bolded with *).

## 4 Discussion

To our knowledge, this study represents the first longitudinal investigation of changes in glymphatic imaging markers following clinical TBI. We focused on two promising and potentially widely available markers: the DTI-ALPS index and ePVS burden. Our key findings are as follows: (1) White matter ePVS burden and ALPS index are significantly correlated with age, as expected. (2) High white matter ePVS burden measured acutely following TBI is significantly associated with a higher number of post-concussion symptoms, particularly memory problems. (3) The increase in the ALPS index from the acute phase (14Day) to the chronic phase (6–12Mon) in patients with mTBI correlates with improvement in sleep measures. Furthermore, incorporating WM-ePVS burden and the ALPS index from the acute visit into patients’ basic clinical information enhances the prediction of chronic memory problems.

The PVS is an anatomical compartment that surrounds brain vascular structures (36). The CSF-filled PVS becomes visible on MRI when enlarged, indicating impaired glymphatic function (31, 36). Enlarged PVS has been recognized as a hallmark of TBI since 2005 (72). Recent studies have reported increased ePVS in moderate-to-severe TBI patients (73), special operations force soldiers with a history of mTBI (41), and Vietnam War veterans with a history of TBI (74). In moderate-to-severe TBI patients, higher ePVS burden is associated with bilateral lesions and worse verbal memory (73). In Vietnam War veterans, high white matter ePVS is associated with poor verbal memory and elevated CSF tau levels, possibly implying impaired waste clearance (74). In another study in Iraq/Afghanistan veterans, higher white matter ePVS was found to be associated with the number of mTBI sustained in the military, poor sleep, and severity of post-concussive symptoms (40). In our mTBI population shortly after injury, no significant differences in ePVS burden compared to the controls were observed, but higher white matter ePVS burden was associated with acute post-injury symptoms, particularly memory problems. These findings align with recent literature on ePVS post-TBI, suggesting that acute changes in the glymphatic system may lead to more severe symptoms. Alternatively, pre-existing conditions may contribute to increased ePVS (36, 75) in mTBI patients, predisposing them to higher post-concussion symptoms and memory problems. Chronic ePVS burden did not correlate with symptoms at 6 -12 months post-injury, possibly indicating active recovery compensating for acute structural glymphatic changes.

Longitudinal changes in the white matter ePVS were not observed in the overall mTBI population or among patients with or without acute memory problems. However, the basal ganglia ePVS burden significantly increased from acute to chronic stages, although no other associations with symptoms were found, either cross-sectionally or longitudinally. The basal ganglia region is commonly associated with ePVS (75), and increased basal ganglia ePVS has been linked to cerebral small vessel diseases in gait disturbance (76), and cognitive decline in early-stage Parkinson’s Disease (77). This suggests vulnerability to vascular and glymphatic changes in this region. The stable nature of white matter ePVS over time aligns with previous reports indicating temporal stability over long follow-up periods (~36 months) (75). Challenges in automatic ePVS segmentation in the basal ganglia, due to reduced T1 weighted signal contrast, may contribute to variability and the lack of correlations with age and symptoms. Previous TBI studies have similarly reported limited findings regarding basal ganglia ePVS (39, 74).

The ALPS index measures diffusion activity specifically along the medullary veins (33), providing a sample of glymphatic activity in the brain. Despite only representing a fraction of overall glymphatic diffusion, DTI-ALPS has gained popularity, particularly due to the widespread availability of diffusion data in MRI of TBI. Reports of ALPS changes post-TBI primarily stem from retrospective studies, mostly showing reduced ALPS index in TBI patients compared to controls (52–54). Lower ALPS index correlates with subarachnoid hemorrhage (52), verbal memory, attention and executive functions (52, 76), and high blood levels of neurofilament light (NfL) chain (53), a typical TBI marker. However, Dai et al. observed increased ALPS in a group of 161 mTBI patients at an acute post-injury time (average of 5.6 days, mostly within 14 days) (55), suggesting dynamic glymphatic activity changes post-TBI. In our study, no significant ALPS differences between control and mTBI groups were found at acute or chronic time points. However, longitudinal changes in the ALPS indexes were significantly associated with concurrent changes in sleep quality over time. Improvement in the sleep conditions correlated with increased glymphatic diffusion activity, indicating a potentially tight connection between sleep and glymphatic function (39, 46, 61, 78, 79). These findings underscore the potential of ALPS index as a promising glymphatic imaging marker linked to changes in sleep conditions following TBI, with sleep possibly mediating TBI outcomes (80–83).

Significant age-related correlations were observed for both white matter ePVS burden and ALPS index, indicating higher ePVS burden and lower ALPS index with older age across both control and mTBI groups. While age-related decline in glymphatic function is well documented (19, 47, 84), our study did not reveal differences in age-related slopes for these glymphatic imaging markers between the control and mTBI groups. Despite TBI being associated with accelerated aging effects, our findings suggest that this effect may not manifest within the early chronic phase post-TBI (6–12 months), indicating a potentially longer timeframe for accelerated aging to develop.

Given that both are considered glymphatic imaging markers, a significant correlation between the ALPS index and white matter ePVS burden was found in the control population, with high ALPS index corresponding to low ePVS burden. However, this relationship was not evident in mTBI patients at either time point. When incorporating glymphatic imaging markers into prediction models for chronic memory problems, acute white matter ePVS and ALPS emerged as significant variables, alongside patient education levels and the presence of CT or MRI findings. Integration of these acute glymphatic imaging markers, derived from clinically available sequences (T1w, T2w, and DTI), may significantly enhance prediction models, suggesting a potential impact of acute glymphatic function on TBI recovery. These findings highlight ePVS burden and ALPS as unique measures of glymphatic function, serving as complementary imaging markers to assess both structural and functional activity aspects of the glymphatic system.

Practical considerations regarding glymphatic imaging markers include ePVS quantification and ALPS estimation. ePVS evaluation traditionally relied on qualitative assessment, limiting accuracy and scalability. Recent studies have adopted automatic segmentation algorithms for larger datasets (32, 45, 85, 86), emphasizing the critical importance of image quality for successful segmentation. Motion artifacts and low signal-to-noise ratio can degrade segmentation quality, leading to potential overestimation of ePVS burden. Manual inspection is essential to ensure data quality. Despite the challenges, ePVS burden remains a robust and stable parameter. Rigorous DTI preprocessing, such as eddy current correction and denoising, improves ALPS index estimation. However, the relationship between sleep cycles and glymphatic activity may contribute to ALPS variability throughout the day, making MRI timing crucial in retrospective designs. Future studies should explore ALPS variability during different times of the day and consider MRI timing in their prospective designs.

Our study has several limitations. First, the control group included both orthopedic patients and healthy controls, potentially introduce trauma-related confounds. Recruitment challenges, such as MRI compatibility issues with orthopedic injuries, made it difficult to exclusively use orthopedic controls for acute imaging, leading us to supplement with healthy controls. While we found no significant differences in glymphatic markers between these groups, trauma-related factors could still influence the results. Second, we did not observe significant symptom improvement in mTBI patients, possibly due to a selection bias favoring those with persistent symptoms. This finding aligns with larger studies like the TRACK-TBI study (10), suggesting that persistent symptoms at one-year post-injury are common. Our study relied on self-reported symptoms, so an important next step is to use more objective memory tests. Third, there are other underlying health conditions (e.g., cardiovascular burden, blood pressure, diabetes), or rehabilitation activities undertaken during study visits may impact the glymphatic function and memory post-TBI. Forth, the relatively small sample size highlights the need for larger studies to better investigate longitudinal glymphatic changes and their role in TBI outcomes. Future research should aim to include a larger and more diverse TBI population to elucidate the role of glymphatic function more accurately in TBI recovery.

## 5 Conclusion

In summary, our longitudinal study using DTI-ALPS and ePVS burden markers reveals significant correlations with age, and high post-TBI ePVS burden is linked to increased symptoms, especially memory problems. An increase in the ALPS index correlates with improved sleep from the acute to chronic stages. Combining these markers with clinical data improves the prediction of chronic memory problems, highlighting their distinct roles in assessing glymphatic structure and activity. Early assessment of glymphatic function could be crucial for understanding TBI recovery and developing targeted interventions to improve patient outcomes.

## Data availability statement

The raw data supporting the conclusions of this article will be made available by the authors, without undue reservation.

## Ethics statement

The studies involving humans were approved by Institutional Review Board of the University of Maryland Baltimore. The studies were conducted in accordance with the local legislation and institutional requirements. The participants provided their written informed consent to participate in this study.

## Author contributions

JZ: Conceptualization, Data curation, Formal analysis, Investigation, Methodology, Project administration, Resources, Software, Supervision, Validation, Visualization, Writing – original draft, Writing – review & editing. PR: Conceptualization, Investigation, Methodology, Writing – review & editing. JL: Data curation, Formal analysis, Writing – review & editing. SR: Formal analysis, Project administration, Resources, Software, Writing – review & editing. RN: Data curation, Project administration, Resources, Writing – review & editing. HC: Formal analysis, Methodology, Writing – review & editing. EW: Methodology, Writing – review & editing. GP: Data curation, Resources, Writing – review & editing. GS: Data curation, Resources, Writing – review & editing. LG: Methodology, Writing – review & editing. ZW: Conceptualization, Methodology, Writing – review & editing. RG: Conceptualization, Funding acquisition, Investigation, Methodology, Project administration, Resources, Supervision, Writing – review & editing. NB: Conceptualization, Funding acquisition, Investigation, Methodology, Resources, Writing – review & editing.

## References

[1] Maas AIR Menon DK Manley GT Abrams M Åkerlund C Andelic N. Traumatic brain injury: progress and challenges in prevention, clinical care, and research. Lancet Neurol. (2022) 21:1004–60. doi: 10.1016/S1474-4422(22)00309-X, PMID: 36183712 PMC10427240

[2] Carroll LJ Cassidy JD Peloso PM Borg J von Holst H Holm L. Prognosis for mild traumatic brain injury: results of the WHO collaborating Centre task force on mild traumatic brain injury. J Rehabil Med Suppl. (2004) 84–105:84–105. doi: 10.1080/16501960410023859, PMID: 15083873

[3] Sours C Rosenberg J Kane R Roys S Zhuo J Shanmuganathan K. Associations between interhemispheric functional connectivity and the automated neuropsychological assessment metrics (ANAM) in civilian mild TBI. Brain Imaging Behav. (2015) 9:190–203. doi: 10.1007/s11682-014-9295-y, PMID: 24557591 PMC4141037

[4] Burrowes SAB Rhodes CS Meeker TJ Greenspan JD Gullapalli RP Seminowicz DA. Decreased grey matter volume in mTBI patients with post-traumatic headache compared to headache-free mTBI patients and healthy controls: a longitudinal MRI study. Brain Imaging Behav. (2019) 14:1651–9. doi: 10.1007/s11682-019-00095-7 PMC7202376 30980274

[5] Zhuo J Jiang L Sours Rhodes C Roys S Shanmuganathan K Chen H. Early stage longitudinal subcortical volumetric changes following mild traumatic brain injury. Brain Inj. (2021) 35:725–33. doi: 10.1080/02699052.2021.1906445, PMID: 33822686 PMC8207827

[6] Drieu A Lanquetin A Prunotto P Gulhan Z Pédron S Vegliante G. Persistent neuroinflammation and behavioural deficits after single mild traumatic brain injury. J Cereb Blood Flow Metab. (2022) 42:2216–29. doi: 10.1177/0271678X221119288, PMID: 35945692 PMC9670002

[7] Trifan G Gattu R Haacke EM Kou Z Benson RR. MR imaging findings in mild traumatic brain injury with persistent neurological impairment. Magn Reson Imaging. (2017) 37:243–51. doi: 10.1016/j.mri.2016.12.009 27939436

[8] Marschner L Schreurs A Lechat B Mogensen J Roebroek A Ahmed T. Single mild traumatic brain injury results in transiently impaired spatial long-term memory and altered search strategies. Behav Brain Res. (2019) 365:222–30. doi: 10.1016/j.bbr.2018.02.040 29499284

[9] Gorman LK Shook BL Becker DP. Traumatic brain injury produces impairments in long-term and recent memory. Brain Res. (1993) 614:29–36. doi: 10.1016/0006-8993(93)91014-J, PMID: 8348320

[10] Machamer J Temkin N Dikmen S Nelson LD Barber J Hwang P. Symptom frequency and persistence in the first year after traumatic brain injury: a TRACK-TBI study. J Neurotrauma. (2022) 39:358–70. doi: 10.1089/neu.2021.0348, PMID: 35078327 PMC8892966

[11] O'Meara ES Kukull WA Sheppard L Bowen JD McCormick WC Teri L. Head injury and risk of Alzheimer’s disease by apolipoprotein E genotype. Am J Epidemiol. (1997) 146:373–84. doi: 10.1093/oxfordjournals.aje.a009290 9290497

[12] Fleminger S Oliver DL Lovestone S Rabe-Hesketh S Giora A. Head injury as a risk factor for Alzheimer’s disease: the evidence 10 years on; a partial replication. J Neurol Neurosurg Psychiatry. (2003) 74:857–62. doi: 10.1136/jnnp.74.7.857, PMID: 12810767 PMC1738550

[13] Li Y Li Y Li X Zhang S Zhao J Zhu X. Head injury as a risk factor for dementia and Alzheimer’s disease: a systematic review and Meta-analysis of 32 observational studies. PLoS One. (2017) 12:e0169650. doi: 10.1371/journal.pone.0169650 28068405 PMC5221805

[14] Brett BL Gardner RC Godbout J Dams-O’Connor K Keene CD. Traumatic brain injury and risk of neurodegenerative disorder. Biol Psychiatry. (2022) 91:498–507. doi: 10.1016/j.biopsych.2021.05.025, PMID: 34364650 PMC8636548

[15] Nedergaard M Goldman SA. Glymphatic failure as a final common pathway to dementia. Science. (2020) 370:50–6. doi: 10.1126/science.abb8739, PMID: 33004510 PMC8186542

[16] Rasmussen MK Mestre H Nedergaard M. The glymphatic pathway in neurological disorders. Lancet Neurol. (2018) 17:1016–24. doi: 10.1016/S1474-4422(18)30318-1, PMID: 30353860 PMC6261373

[17] Iliff JJ Wang M Liao Y Plogg BA Peng W Gundersen GA. A paravascular pathway facilitates CSF flow through the brain parenchyma and the clearance of interstitial solutes, including amyloid β. Sci Transl Med. (2012) 4:147ra111. doi: 10.1126/scitranslmed.3003748, PMID: 22896675 PMC3551275

[18] Nedergaard M. Neuroscience. Garbage truck of the brain. Science. (2013) 340:1529–30. doi: 10.1126/science.1240514, PMID: 23812703 PMC3749839

[19] Benveniste H Liu X Koundal S Sanggaard S Lee H Wardlaw J. The Glymphatic system and waste clearance with brain aging: a review. Gerontology. (2019) 65:106–19. doi: 10.1159/000490349, PMID: 29996134 PMC6329683

[20] Reddy OC van der Werf YD. The sleeping brain: harnessing the power of the Glymphatic system through lifestyle choices. Brain Sci. (2020) 10:1–16. doi: 10.3390/brainsci10110868 PMC7698404 33212927

[21] Xie L Kang H Xu Q Chen MJ Liao Y Thiyagarajan M. Sleep drives metabolite clearance from the adult brain. Science. (2013) 342:373–7. doi: 10.1126/science.1241224, PMID: 24136970 PMC3880190

[22] Iliff JJ Chen MJ Plog BA Zeppenfeld DM Soltero M Yang L. Impairment of glymphatic pathway function promotes tau pathology after traumatic brain injury. J Neurosci. (2014) 34:16180–93. doi: 10.1523/JNEUROSCI.3020-14.2014, PMID: 25471560 PMC4252540

[23] Peters ME Lyketsos CG. The glymphatic system’s role in traumatic brain injury-related neurodegeneration. Mol Psychiatry. (2023) 28:2707–15. doi: 10.1038/S41380-023-02070-7, PMID: 37185960

[24] Lee Y Choi Y Park EJ Kwon S Kim H Lee JY. Improvement of glymphatic-lymphatic drainage of beta-amyloid by focused ultrasound in Alzheimer’s disease model. Sci Rep. (2020) 10:16144. doi: 10.1038/s41598-020-73151-8 32999351 PMC7527457

[25] Kashyap S Brazdzionis J Savla P Berry JA Farr S Patchana T. Osteopathic manipulative treatment to optimize the Glymphatic environment in severe traumatic brain injury measured with optic nerve sheath diameter, intracranial pressure monitoring, and neurological pupil index. Cureus. (2021) 13:e13823. doi: 10.7759/cureus.13823 33859888 PMC8038899

[26] Guernsey DT Leder A Yao S. Resolution of concussion symptoms after osteopathic manipulative treatment: a Case report. J Am Osteopath Assoc. (2016) 116:e13–7. doi: 10.7556/jaoa.2016.036, PMID: 26927914

[27] Eide PK Mariussen E Uggerud H Pripp AH Lashkarivand A Hassel B. Clinical application of intrathecal gadobutrol for assessment of cerebrospinal fluid tracer clearance to blood. JCI Insight. (2021) 6:147063. doi: 10.1172/jci.insight.147063, PMID: 33822769 PMC8262318

[28] Ringstad G Vatnehol SAS Eide PK. Glymphatic MRI in idiopathic normal pressure hydrocephalus. Brain. (2017) 140:2691–705. doi: 10.1093/brain/awx191, PMID: 28969373 PMC5841149

[29] Boyd ED Kaur J Ding G Chopp M Jiang Q. Clinical magnetic resonance imaging evaluation of glymphatic function. NMR Biomed. (2024):e5132. doi: 10.1002/NBM.5132, PMID: 38465514

[30] Klostranec JM Vucevic D Bhatia KD Kortman HGJ Krings T Murphy KP. Current concepts in intracranial interstitial fluid transport and the Glymphatic system: part I-anatomy and physiology. Radiology. (2021) 301:502–14. doi: 10.1148/radiol.2021202043 34665028

[31] Ramirez J Berezuk C McNeely AA Gao F McLaurin JA Black SE. Imaging the perivascular space as a potential biomarker of neurovascular and neurodegenerative diseases. Cell Mol Neurobiol. (2016) 36:289–99. doi: 10.1007/s10571-016-0343-6 26993511 PMC11482437

[32] Sepehrband F Barisano G Sheikh-Bahaei N Cabeen RP Choupan J Law M. Image processing approaches to enhance perivascular space visibility and quantification using MRI. Sci Rep. (2019) 9:12351. doi: 10.1038/s41598-019-48910-x, PMID: 31451792 PMC6710285

[33] Taoka T Masutani Y Kawai H Nakane T Matsuoka K Yasuno F. Evaluation of glymphatic system activity with the diffusion MR technique: diffusion tensor image analysis along the perivascular space (DTI-ALPS) in Alzheimer’s disease cases. Jpn J Radiol. (2017) 35:172–8. doi: 10.1007/s11604-017-0617-z 28197821

[34] Liang T Chang F Huang Z Peng D Zhou X Liu W. Evaluation of glymphatic system activity by diffusion tensor image analysis along the perivascular space (DTI-ALPS) in dementia patients. Br J Radiol. (2023) 96:20220315. doi: 10.1259/bjr.20220315, PMID: 37066824 PMC10230386

[35] Wardlaw JM Smith EE Biessels GJ Cordonnier C Fazekas F Frayne R. Neuroimaging standards for research into small vessel disease and its contribution to ageing and neurodegeneration. Lancet Neurol. (2013) 12:822–38. doi: 10.1016/S1474-4422(13)70124-8, PMID: 23867200 PMC3714437

[36] Wardlaw JM Benveniste H Nedergaard M Zlokovic BV Mestre H Lee H. Perivascular spaces in the brain: anatomy, physiology and pathology. Nat Rev Neurol. (2020) 16:137–53. doi: 10.1038/s41582-020-0312-z, PMID: 32094487

[37] Zhu YC Dufouil C Mazoyer B Soumaré A Ricolfi F Tzourio C. Frequency and location of dilated Virchow-Robin spaces in elderly people: a population-based 3D MR imaging study. AJNR Am J Neuroradiol. (2011) 32:709–13. doi: 10.3174/ajnr.A2366, PMID: 21349956 PMC7965873

[38] Francis F Ballerini L Wardlaw JM. Perivascular spaces and their associations with risk factors, clinical disorders and neuroimaging features: a systematic review and meta-analysis. Int J Stroke. (2019) 14:359–71. doi: 10.1177/1747493019830321, PMID: 30762496

[39] Opel RA Christy A Boespflug EL Weymann KB Case B Pollock JM. Effects of traumatic brain injury on sleep and enlarged perivascular spaces. J Cereb Blood Flow Metab. (2019) 39:2258–67. doi: 10.1177/0271678X18791632 30092696 PMC6827121

[40] Piantino J Schwartz DL Luther M Newgard C Silbert L Raskind M. Link between mild traumatic brain injury, poor sleep, and magnetic resonance imaging: visible perivascular spaces in veterans. J Neurotrauma. (2021) 38:2391–9. doi: 10.1089/neu.2020.7447, PMID: 33599176 PMC8390772

[41] Powell JR Zong X Weinstein JM DeLellis SM Kane SF Means GE. Mild traumatic brain injury and career stage associate with visible perivascular spaces in special operations forces soldiers. Ann Biomed Eng. (2024). doi: 10.1007/S10439-024-03468-2, PMID: 38396272

[42] Zhu YC Dufouil C Soumaré A Mazoyer B Chabriat H Tzourio C. High degree of dilated Virchow-Robin spaces on MRI is associated with increased risk of dementia. J Alzheimers Dis. (2010) 22:663–72. doi: 10.3233/JAD-2010-100378, PMID: 20847444

[43] Weller RO Hawkes CA Kalaria RN Werring DJ Carare RO. White matter changes in dementia: role of impaired drainage of interstitial fluid. Brain Pathol. (2015) 25:63–78. doi: 10.1111/bpa.12218, PMID: 25521178 PMC8028907

[44] Berezuk C Ramirez J Gao F Scott CJM Huroy M Swartz RH. Virchow-Robin spaces: correlations with polysomnography-derived sleep parameters. Sleep. (2015) 38:853. doi: 10.5665/sleep.4726 26163465 PMC4434551

[45] Shih NC Barisano G Lincoln KD Mack WJ Sepehrband F Choupan J. Effects of sleep on brain perivascular space in a cognitively healthy population. Sleep Med. (2023) 111:170–9. doi: 10.1016/j.sleep.2023.09.024, PMID: 37782994 PMC10591884

[46] Zhuo J Raghavan P Shao M Roys S Liang X Tchoquessi RLN. Automatic quantification of enlarged perivascular space in patients with traumatic brain injury using super-resolution of T2-weighted images. J Neurotrauma. (2024) 41:407–19. doi: 10.1089/neu.2023.0082, PMID: 37950721 PMC10837035

[47] Hsiao WC Chang HI Hsu SW Lee CC Huang SH Cheng CH. Association of Cognition and Brain Reserve in aging and Glymphatic function using diffusion tensor image-along the perivascular space (DTI-ALPS). Neuroscience. (2023) 524:11–20. doi: 10.1016/j.neuroscience.2023.04.004, PMID: 37030632

[48] Lee HJ Lee DA Shin KJ Park KM. Glymphatic system dysfunction in obstructive sleep apnea evidenced by DTI-ALPS. Sleep Med. (2022) 89:176–81. doi: 10.1016/j.sleep.2021.12.013, PMID: 35030357

[49] Lee DA Lee HJ Park KM. Glymphatic dysfunction in isolated REM sleep behavior disorder. Acta Neurol Scand. (2022) 145:464–70. doi: 10.1111/ane.13573, PMID: 34918348

[50] Ota M Sato N Nakaya M Shigemoto Y Kimura Y Chiba E. Relationships between the deposition of amyloid-β and tau protein and Glymphatic system activity in Alzheimer’s disease: diffusion tensor image study. J Alzheimers Dis. (2022) 90:295–303. doi: 10.3233/JAD-220534 36120782

[51] Chang HI Huang CW Hsu SW Huang SH Lin KJ Ho TY. Gray matter reserve determines glymphatic system function in young-onset Alzheimer’s disease: evidenced by DTI-ALPS and compared with age-matched controls. Psychiatry Clin Neurosci. (2023) 77:401–9. doi: 10.1111/pcn.13557, PMID: 37097074 PMC11488612

[52] Park JH Bae YJ Kim JS Jung WS Choi JW Roh TH. Glymphatic system evaluation using diffusion tensor imaging in patients with traumatic brain injury. Neuroradiology. (2023) 65:551–7. doi: 10.1007/s00234-022-03073-x, PMID: 36274107

[53] Butler T Zhou L Ozsahin I Wang XH Garetti J Zetterberg H. Glymphatic clearance estimated using diffusion tensor imaging along perivascular spaces is reduced after traumatic brain injury and correlates with plasma neurofilament light, a biomarker of injury severity. Brain Commun. (2023) 5:fcad134. doi: 10.1093/braincomms/fcad134 37188222 PMC10176239

[54] Yang DX Sun Z Yu MM Zou QQ Li PY Zhang JK. Associations of MRI-derived Glymphatic system impairment with global white matter damage and cognitive impairment in mild traumatic brain injury: a DTI-ALPS study. J Magn Reson Imaging. (2024) 59:639–47. doi: 10.1002/jmri.28797 37276070

[55] Dai Z Yang Z Li Z Li M Sun H Zhuang Z. Increased glymphatic system activity in patients with mild traumatic brain injury. Front Neurol. (2023) 14:1148878. doi: 10.3389/fneur.2023.1148878, PMID: 37251219 PMC10213560

[56] Déry J Ouellet B de Guise É Bussières ÈL Lamontagne ME. Prognostic factors for persistent symptoms in adults with mild traumatic brain injury: an overview of systematic reviews. Syst Rev. (2023) 12:127. doi: 10.1186/s13643-023-02284-4, PMID: 37468999 PMC10357711

[57] King N. Permanent post concussion symptoms after mild head injury: a systematic review of age and gender factors. Neuro Rehabil. (2014) 34:741–8. doi: 10.3233/NRE-141072, PMID: 24796434

[58] King NS Crawford S Wenden FJ Moss NEG Wade DT. The Rivermead post concussion symptoms questionnaire: a measure of symptoms commonly experienced after head injury and its reliability. J Neurol. (1995) 242:587–92. doi: 10.1007/BF00868811 8551320

[59] Buysse DJ Reynolds CF Monk TH Berman SR Kupfer DJ. The Pittsburgh sleep quality index: a new instrument for psychiatric practice and research. Psychiatry Res. (1989) 28:193–213. doi: 10.1016/0165-1781(89)90047-4, PMID: 2748771

[60] Kalmbach DA Conroy DA Falk H Rao V Roy D Peters ME. Poor sleep is linked to impeded recovery from traumatic brain injury. Sleep. (2018) 41:zsy147. doi: 10.1093/sleep/zsy147, PMID: 30053263 PMC6890523

[61] Tang S Sours Rhodes C Jiang L Chen H Roys S Badjatia N. Association between sleep disturbances at subacute stage of mild traumatic brain injury and long-term outcomes. Neurotrauma Rep. (2022) 3:276–85. doi: 10.1089/neur.2022.0004, PMID: 35982983 PMC9380873

[62] Glasser MF Sotiropoulos SN Wilson JA Coalson TS Fischl B Andersson JL. The minimal preprocessing pipelines for the human connectome project. NeuroImage. (2013) 80:105–24. doi: 10.1016/j.neuroimage.2013.04.127, PMID: 23668970 PMC3720813

[63] Tournier JD Smith R Raffelt D Tabbara R Dhollander T Pietsch M. MRtrix3: a fast, flexible and open software framework for medical image processing and visualisation. NeuroImage. (2019) 202:116137. doi: 10.1016/j.neuroimage.2019.116137, PMID: 31473352

[64] Veraart J Novikov DS Christiaens D Ades-aron B Sijbers J Fieremans E. Denoising of diffusion MRI using random matrix theory. NeuroImage. (2016) 142:394–406. doi: 10.1016/j.neuroimage.2016.08.016, PMID: 27523449 PMC5159209

[65] Andersson JLR Sotiropoulos SN. An integrated approach to correction for off-resonance effects and subject movement in diffusion MR imaging. NeuroImage. (2016) 125:1063–78. doi: 10.1016/j.neuroimage.2015.10.019, PMID: 26481672 PMC4692656

[66] Tustison NJ Avants BB Cook PA Yuanjie Zheng Egan A Yushkevich PA. N4ITK: improved N3 bias correction. IEEE Trans Med Imaging. (2010) 29:1310–20. doi: 10.1109/TMI.2010.2046908, PMID: 20378467 PMC3071855

[67] Zhuo J Xu S Proctor JL Mullins RJ Simon JZ Fiskum G. Diffusion kurtosis as an in vivo imaging marker for reactive astrogliosis in traumatic brain injury. NeuroImage. (2012) 59:467–77. doi: 10.1016/j.neuroimage.2011.07.050, PMID: 21835250 PMC3614502

[68] Stokum JA Sours C Zhuo J Kane R Shanmuganathan K Gullapalli RP. A longitudinal evaluation of diffusion kurtosis imaging in patients with mild traumatic brain injury. Brain Inj. (2015) 29:47–57. doi: 10.3109/02699052.2014.947628, PMID: 25259786

[69] Tatekawa H Matsushita S Ueda D Takita H Horiuchi D Atsukawa N. Improved reproducibility of diffusion tensor image analysis along the perivascular space (DTI-ALPS) index: an analysis of reorientation technique of the OASIS-3 dataset. Jpn J Radiol. (2023) 41:393–400. doi: 10.1007/s11604-022-01370-2, PMID: 36472803 PMC10066136

[70] Jenkinson M Beckmann CF Behrens TEJ Woolrich MW Smith SM. FSL. NeuroImage. (2012) 62:782–90. doi: 10.1016/j.neuroimage.2011.09.015 21979382

[71] Avants BB Epstein CL Grossman M Gee JC. Symmetric diffeomorphic image registration with cross-correlation: evaluating automated labeling of elderly and neurodegenerative brain. Med Image Anal. (2008) 12:26–41. doi: 10.1016/j.media.2007.06.004, PMID: 17659998 PMC2276735

[72] Inglese M Bomsztyk E Gonen O Mannon LJ Grossman RI Rusinek H. Dilated perivascular spaces: hallmarks of mild traumatic brain injury. AJNR Am J Neuroradiol. (2005) 26:719–24. PMID: 15814911 PMC7977096

[73] Hicks A Sinclair B Shultz SR Pham W Silbert LC Schwartz DL. Associations of enlarged perivascular spaces with brain lesions, brain age, and clinical outcomes in chronic traumatic brain injury. Neurology. (2023) 101:e63–73. doi: 10.1212/WNL.0000000000207370, PMID: 37156615 PMC10351302

[74] Wang ML Yang DX Sun Z Li WB Zou QQ Li PY. MRI-visible perivascular spaces associated with cognitive impairment in military veterans with traumatic brain injury mediated by CSF P-tau. Front Psych. (2022) 13:921203. doi: 10.3389/fpsyt.2022.921203 PMC9299379 35873253

[75] Okar SV Hu F Shinohara RT Beck ES Reich DS Ineichen BV. The etiology and evolution of magnetic resonance imaging-visible perivascular spaces: systematic review and meta-analysis. Front Neurosci. (2023) 17:1038011. doi: 10.3389/fnins.2023.1038011, PMID: 37065926 PMC10098201

[76] Yang S Li X Hu W Qin W Yang L. Enlarged perivascular spaces in the basal ganglia independently related to gait disturbances in older people with cerebral small vessel diseases. Front Aging Neurosci. (2022) 14:833702. doi: 10.3389/fnagi.2022.833702 35813945 PMC9257267

[77] Shen T Yue Y Zhao S Xie J Chen Y Tian J. The role of brain perivascular space burden in early-stage Parkinson’s disease. NPJ Parkinsons Dis. (2021) 7:12. doi: 10.1038/s41531-021-00155-0, PMID: 33547311 PMC7864928

[78] Piantino J Lim MM Newgard CD Iliff J. Linking traumatic brain injury, sleep disruption and post-traumatic headache: a potential role for Glymphatic pathway dysfunction. Curr Pain Headache Rep. (2019) 23:62. doi: 10.1007/s11916-019-0799-4 31359173

[79] Piantino JA Iliff JJ Lim MM. The bidirectional link between sleep disturbances and traumatic brain injury symptoms: a role for Glymphatic dysfunction? Biol Psychiatry. (2022) 91:478–87. doi: 10.1016/j.biopsych.2021.06.025, PMID: 34481662 PMC8758801

[80] Wickwire EM Williams SG Roth T Capaldi VF Jaffe M Moline M. Sleep, sleep disorders, and mild traumatic brain injury. What we know and what we need to know: findings from a National Working Group. Neurotherapeutics. (2016) 13:403–17. doi: 10.1007/s13311-016-0429-3, PMID: 27002812 PMC4824019

[81] Wickwire E Albrecht JS Capaldi VF II Jain S Gardner RC Smith MT. Association between insomnia and mental health and neurocognitive outcomes following traumatic brain injury. J Neurotrauma. (2023) 40:2376–85. doi: 10.1089/NEU.2023.0009, PMID: 37463057 PMC13588594

[82] Wickwire EM Schnyer DM Germain A Smith MT Williams SG Lettieri CJ. Sleep, sleep disorders, and circadian health following mild traumatic brain injury in adults: review and research agenda. J Neurotrauma. (2018) 35:2615–31. doi: 10.1089/neu.2017.5243, PMID: 29877132 PMC6239093

[83] Wickwire EM Albrecht JS Griffin NR Schnyer DM Yue JK Markowitz AJ. Sleep disturbances precede depressive symptomatology following traumatic brain injury. Curr Neurobiol. (2019) 10:49–55. PMID: 34040318 PMC8148630

[84] Dai Z Yang Z Chen X Zheng W Zhuang Z Liao Y. The aging of glymphatic system in human brain and its correlation with brain charts and neuropsychological functioning. Cereb Cortex. (2023) 33:7896–903. doi: 10.1093/cercor/bhad086, PMID: 36928180

[85] Lynch KM Sepehrband F Toga AW Choupan J. Brain perivascular space imaging across the human lifespan. NeuroImage. (2023) 271:120009. doi: 10.1016/j.neuroimage.2023.120009, PMID: 36907282 PMC10185227

[86] Ballerini L Lovreglio R Valdés Hernández MC Ramirez J MacIntosh BJ Black SE. Perivascular spaces segmentation in brain MRI using optimal 3D filtering. Sci Rep. (2018) 8:2132. doi: 10.1038/s41598-018-19781-5, PMID: 29391404 PMC5794857

